# Supplementary figures and images for: THC and sperm: Impact on fertilization capability, pre-implantation in vitro development and epigenetic modifications
Source: PLoS One. 2024 Mar 27;19(3):e0298697. doi: 10.1371/journal.pone.0298697 (PMC10971525; doi:10.1371/journal.pone.0298697)

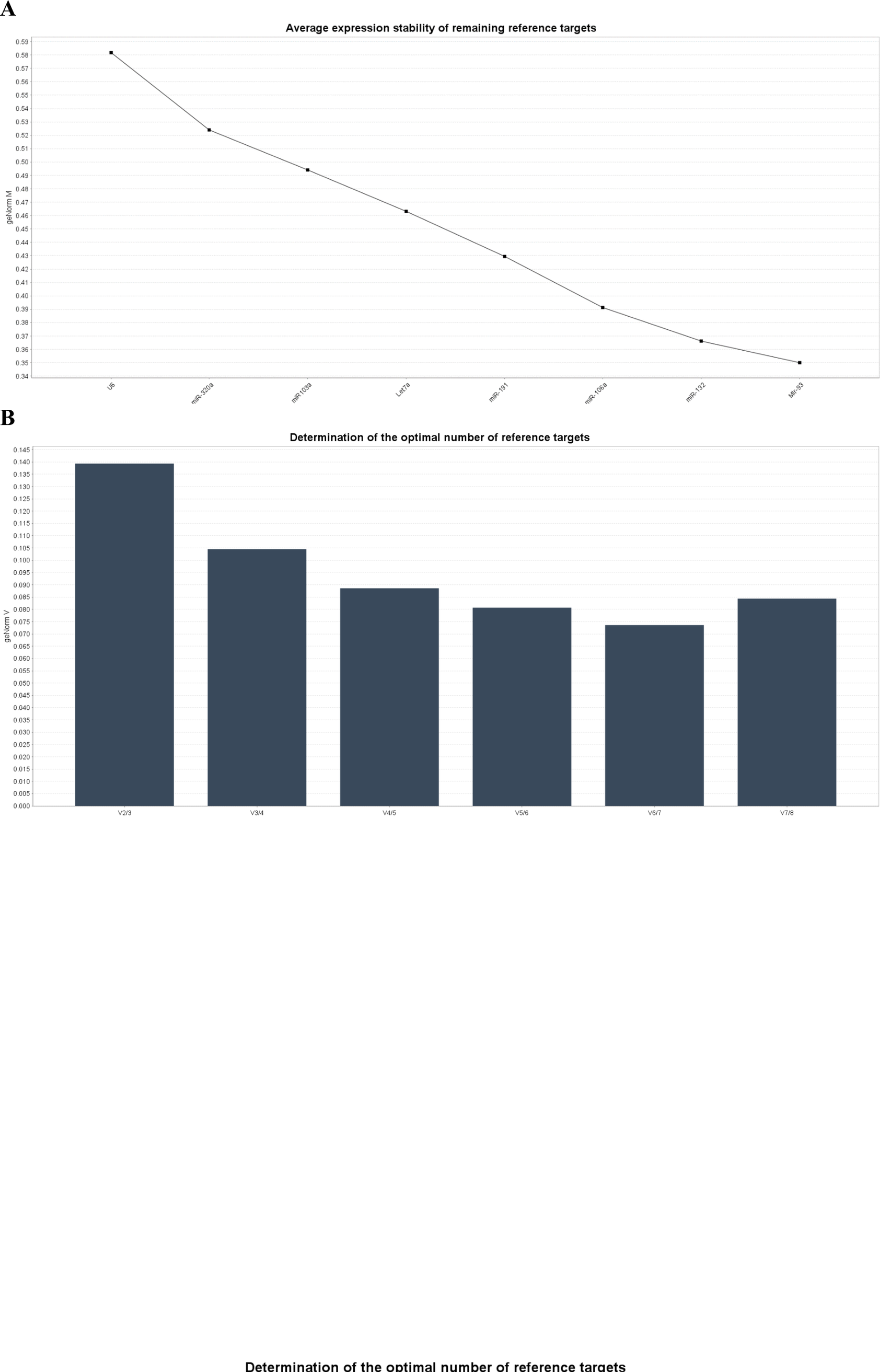

Supplement: S1 Fig — (A) Average stability of 8 candidate genes: U6, miRNA-320a, miRNA-103a, Let7a, miRNA191, miRNA-106a, miRNA-132, and miRNA-93. (B) Determination of the optimal number of reference ttargets with a V vale set at 0.15, showing that 2/3-7/8 reference genes can be used to obtain the approriate reference value. (TIF) [file pone.0298697.s001.tif]
